# Capturing the Experience: How Digital Media Affects Memory Retention in Museum Education

**DOI:** 10.3390/bs15091247

**Published:** 2025-09-12

**Authors:** Serkan Say, Serdar Akbulut, İsmail Yavuz Öztürk

**Affiliations:** 1 Faculty of Education, Department of Basic Education, Mersin University, Mersin 33110, Türkiye; serkansay@mersin.edu.tr; 2 Faculty of Education, Department of Turkish Language and Social Sciences Education, Manisa Celal Bayar University, Manisa 45140, Türkiye; 3 Faculty of Education, Department of Turkish Language and Social Sciences Education, Mersin University, Mersin 33110, Türkiye; iyavuzozturk@mersin.edu.tr

**Keywords:** cognitive offloading, digital media, memory retention

## Abstract

This study investigates the effects of digital media usage, specifically photo-taking and video recording, on memory retention in the context of museum education. Utilizing a quasi-experimental design, this research involved three groups, each exposed to different conditions: observation without media use, photo-taking, and video recording. A total of 120 university students who participated in the study were divided randomly into groups balanced by working memory capacity. Immediate and delayed recall tests were conducted to assess short-term memory and long-term retention. The results reveal that participants who merely observed the objects exhibited considerably better memory performance compared to those who used digital media. This result is consistent with the cognitive offloading hypothesis and suggests that digital devices weaken memory encoding processes by reducing individuals’ internal cognitive resources. The video-recording group exhibited the lowest performance due to the need for sustained attention and increased cognitive load. The photographing group, despite performing lower in the short-term memory test, showed less decline in the long-term memory test than the other groups. This suggests that photographs may serve as a cue in the retrieval process. The research findings reveal that digital media use can have both supportive and disruptive effects in educational environments. In this context, it is important for educators and museum designers to develop strategies that will consciously direct the use of digital tools.

## 1. Introduction

In recent years, due to the widespread use of digital technologies and their profound effects on memory and cognitive processes (Finley et al., 2018), the act of taking photographs in museums and cultural institutions has become an almost automatic behavior. The increasing use of smartphones and other devices as external memory aids has led to the study of the photograph–distortion effect. This phenomenon suggests that taking photographs may disrupt memory encoding and retrieval processes, inhibiting memory retention that occurs through natural cognitive processes (Henkel, 2014; Soares & Storm, 2018). These effects are particularly important in museum education, as museum exhibits are meticulously designed to increase visitor engagement, reflection, and memory consolidation. However, growing evidence suggests that taking photographs may undermine these educational experiences by distracting and reducing cognitive engagement (Diehl et al., 2016; Lurie & Westerman, 2021).

The photographing–distortion effect can be explained by the concept of cognitive offloading, which refers to transferring cognitive load to external devices. This concept refers to the reduction in cognitive load on internal memory systems when external devices such as smartphones are used to store information (Risko & Gilbert, 2016). This proposition is consistent with the transactive memory theory (Wegner, 1987), according to which individuals divide memory tasks between themselves and external sources, reducing the need for internal memory. For example, Henkel (2014) found that participants who took photographs during a museum visit remembered fewer details than those who only observed. Moreover, research indicated that taking photographs competed with attentional resources that should be allocated to deep cognitive processing (Niforatos et al., 2017). Furthermore, cognitive load theory (Chandler & Sweller, 1991) emphasizes the importance of managing working memory capacity to support effective learning. In educational environments where experiential learning is central, such as museums, performing tasks such as having the experience and taking photos or videos simultaneously can overload cognitive resources. As Sweller (1988, 2010) noted, when students divide their attention between interacting with the exhibits and using recording devices, their cognitive performance may deteriorate. In addition, the redundancy effect, which can be considered within the scope of Cognitive Load Theory, should also be taken note of at this point. The redundancy effect occurs when different sources provide the *same* (repeating information) or *unnecessary* (information unrelated to the learning content) information (Chandler & Sweller, 1991) and create additional load on memory, hampering learning, and it should therefore be eliminated (Trypke et al., 2023). Accordingly, in addition to viewing the objects both visually and through the smartphone screen, the stimulus of camera application and other stimuli around the objects may have the potential to provide the same or unnecessary information flow from different sources and create additional load on students’ working memories. Furthermore, guided observation practices (giving structured prompts that direct learners’ attention to specific elements) have been shown to reduce unnecessary cognitive load (Kirschner et al., 2006; Mayer, 2005). In the museum context, this type of structured observation promotes both recall and conceptual comprehension by enabling the effective use of working memory resources (Moreno & Mayer, 2007).

Even though growing research has focused on the effects of taking photographs, video recording leads to additional complexities. Video recording requires a longer interaction with the device, which may result in greater cognitive impairments than taking photographs (Diehl et al., 2016). While taking photographs has sometimes been shown to enhance recall of visual details (Barasch et al., 2017; Tan & Jiang, 2018), video recording—due to the need for sustained attention—incurs a higher cognitive load (Murphy, 2023). In this context, the effects of video recording, particularly in educational settings, have not been sufficiently investigated compared to taking photographs. This gap highlights the need for a more nuanced understanding of how video recording impacts memory processes as the integration of digital tools into learning experiences increases.

Given the complex relationship between digital media use, attention, and memory, it is crucial to examine the effects of behaviors such as photography and video recording on both short-term and long-term memory in educational contexts. While existing research has largely focused on the effects of photography (Barasch et al., 2017; Fawns, 2022; Henkel, 2014; Soares & Storm, 2018; Tamir et al., 2018), the cognitive effects of video recording, especially when compared to photography, have not been studied sufficiently. Understanding how storing or deleting content via photography or video affects memory retention is vital for developing strategies to minimize cognitive load while optimizing learning. As digital technologies such as photography and video increasingly become more prevalent in daily life and educational environments, examining the cognitive effects of these tools is critical to improving learning outcomes. Although there are extensive studies on the effects of taking photos, the effect of video recording on memory processes in the context of museum education is not highlighted (Diehl & Zauberman, 2022; Wong & Lim, 2023). Video recording creates a specific cognitive load on museum-based learning objectives because it requires continuity and intense attention. Considering the video sharing habits that are becoming more common today (especially in social media) (Diehl et al., 2016; Kislinger & Kotrschal, 2021; Weilenmann et al., 2013), examining the effect of video recording on memory processes is considered important in terms of determining how digital devices shape memory and meaning-making processes in museums. This study aims to make valuable contributions to the broader debate on digital media use and cognitive offloading by investigating the effects of photography and video recording on memory processes in the context of museum education.

### Theoretical Framework

The relationship between digital technologies and memory processes has recently been a topic of growing interest among scholars, particularly in the context of education. The increasing integration of digital devices such as smartphones and cameras into everyday life requires a careful examination of their effects on memory, especially in environments that encourage learning and thinking, such as museums. Much of the research in this area has focused on the “photographing–distortion effect,” which suggests that taking photographs disrupts natural memory processes by disrupting memory encoding and retrieval processes (Henkel, 2014; Soares & Storm, 2018). In the museum context, memory is not merely a process of recalling factual details; it is also a fundamental cognitive function for deeper cultural interpretation, critical thinking, and aesthetic evaluation (Falk & Dierking, 2013; Hein, 1998). Memory persistence from a pedagogical perspective paves the way for visitors to place their experiences within historical and cultural narratives, thus establishing meaning and developing cultural awareness. Thus, the question of how digital tools mediate memory processes in museum education is marked as a critical research area. This effect is particularly important in museum education, which is designed for active participation, thinking, and knowledge consolidation. However, the emergence of pervasive digital technologies suggests that these devices can negatively impact the learning experience due to their potential to distract users while documenting rather than allowing them to focus on the material (Lurie & Westerman, 2021).

Cognitive Offloading and Memory Encoding: Central to the photo–distortion effect is the concept of “cognitive offloading,” the externalization of cognitive processes. This concept suggests that individuals offload cognitive tasks, such as memory storage, to devices such as smartphones, reducing the mental load required to recall and process information (Risko & Gilbert, 2016). The increased use of smartphones as an external memory aid may reduce the use of individuals’ internal cognitive resources, which may interfere with deep learning and long-term memory consolidation. While digital technologies serve practical purposes, when these devices replace active memory processes, they may undermine individuals’ ability to process and internalize complex or detailed information (Risko et al., 2019).

In educational contexts such as museums, where deep cognitive engagement is expected, the reliance on processes involving digital documentation, such as taking photographs, can reduce attention to the material on display. This effect is linked to working memory, which is a cognitive system that temporarily holds and processes information during tasks such as problem solving, comprehension, and learning (Baddeley, 2000). Cognitive offloading through media use can cause individuals to engage less with the material, leading to lower working memory engagement and ultimately impaired learning outcomes. Studies such as Sparrow et al. (2011) and Greener (2017) have shown that reliance on external memory aids affects long-term retrieval by shifting away from active memory encoding.

Transactive memory theory (Wegner, 1987) extends this concept further, suggesting that individuals divide memory tasks between themselves and external sources. Transactive memory systems are shared not only among people in group settings, but also among devices such as smartphones and cameras. In the context of museum education, this means that visitors can rely on their devices to capture and store their experiences, thus reducing the cognitive effort required to remember the material on display. While this can free up cognitive resources for other tasks, it can also result in superficial engagement with the material, as internal memory processes are delegated to external devices.

Photo-Taking–Impairment Effect: This effect, referring to photographs interfering with memory processes, has been at the center of studies investigating how digital technologies affect memory processes. First studied by Henkel (2014), this effect suggests that taking photos during an experience can reduce a person’s ability to remember details from that experience. Henkel’s study of participants on a guided museum tour found that those who took photos remembered fewer details about objects than those who simply observed the exhibits. This finding suggests that the act of taking photos may interrupt cognitive processes involved in encoding visual and contextual information, leading to poorer memory consolidation. When visitors turn their attention to capturing images, they may miss the opportunity to engage deeply with the exhibit content (Henkel, 2014).

Extending Henkel’s findings, several studies have examined the broader contexts of the photo-taking impairment effect. Soares and Storm (2018) investigated the effects of conscious cognitive offloading by taking photos on recall experiences and found that participants who used photos as an external memory aid performed worse on memory tests compared to those who did not take photos. This research highlights that even when individuals consciously delegate memory tasks to external devices, they may unintentionally diminish their engagement with the material. The cognitive resources devoted to the process of capturing and managing images may undermine the attention required for deep learning, reinforcing that taking images impairs memory retention (Soares & Storm, 2022).

Despite the noted negative effects, some research asserts that taking photos can improve memory under certain conditions. For instance, Barasch et al. (2017) found that those who took photos with the intention of remembering visual details developed better memory for those details. However, this benefit appears to be limited to superficial types of visual information only and does not extend to deeper or contextual elements of an experience. This suggests that taking photos may improve memory for some details but generally impairs overall memory of an event because cognitive resources are divided between documenting the experience and directly engaging with it.

Video Recording and Cognitive Load: The cognitive effects of video recording are less researched compared to the effects of taking photos. While taking photos is a momentary action, video recording is a more cognitively complex task as it requires continuous interaction with the device. Controlling the camera, keeping the frame steady, and focusing on the ongoing action during video recording imposes a higher cognitive load compared to taking a single photo (Barasch et al., 2017; Wong & Lim, 2023). This enhanced cognitive demand coincides with ‘cognitive load theory’, which emphasizes the limited capacity of working memory (Chandler & Sweller, 1991). According to the theory, processing too much information at once can lead to cognitive overload and thus decreased memory performance.

Sweller (2010) divides cognitive load into three categories: intrinsic, extrinsic, and germane load. Within the realm of video recording, the extrinsic load associated with the use of the recording device competes with intrinsic cognitive processes such as understanding and remembering learning material such as exhibits. Video recording requires sustained attention, reducing the cognitive resources available to process the material. Such divided attention can lead to poorer memory encoding and thus less effective learning outcomes (Sweller et al., 2011).

Diehl et al.’s (2016) study demonstrates the varying effects of video recording and photography on cognitive performance. The study results show that both actions have cognitive costs, but video recording may lead to more significant memory impairments due to the need for sustained attention. Like photography, video recording can also strain cognitive resources in educational environments such as museums, reducing the depth of the learning experience. Understanding these differences between photography and video recording is of great importance for educators and museum staff who want to design learning environments that maximize cognitive engagement and at the same time minimizing the negative effects of media use.

## 2. Research Methods

This study used a quasi-experimental method to investigate the effects of digital media use on memory in the context of museum education (Creswell & Creswell, 2017). This method allowed causal relationships between variables to be examined by applying different interventions. These interventions, designed to evaluate the effects of media use such as photography and video recording on both immediate and long-term memory, allowed for a systematic analysis of the effects on learning and memory processes.

### 2.1. Participants

Within the scope of the study, as 3 different procedures were applied in order to evaluate the effects of different learning strategies during the museum tour, we planned to create 3 separate groups. Initially, 120 volunteer university students (between the ages of 18 and 25) were selected using the convenience sampling method. In order to ensure a balance of working memory capacity across the groups formed, a working memory capacity test was applied to all the participants. This test was preferred because the primary condition for learners to be able to store newly encountered information in long-term memory and to be able to recall it later is their working memory capacity (Chandler & Sweller, 1991; Unsworth et al., 2013). According to the test results, a total of 60 people were selected in 3 groups with close averages in terms of memory capacities and a low probability of interacting with each other. This process provides a more accurate measurement of the effects of different procedures by creating a homogeneous structure among the groups.

Eventually, three separate groups were formed with the 60 people determined. Accordingly, the first group would only observe the objects and would not take photos or videos; the second group would only be able to take photos of the same objects; and the third group would only be able to record videos of the same objects in the same museum. These groups were formed based on the students’ preferences and aimed to compare different learning and experiencing styles in line with these preferences. Thus, each participant participated in the museum trip with a learning method based on their own preference. Another reason for offering participants the right to choose is that there are findings that suggest that giving individuals the right to choose increases their awareness, active participation and general well-being levels (Langer & Rodin, 2004), in addition to their intrinsic motivation, effort, task performance and perceived competence levels (Patall et al., 2008).

Considering this distribution, the number of participants was determined as 60 and three groups were selected according to the test results and included in the study. To ensure the internal validity of the study and isolate the effects of the independent variable (digital media use) on the dependent variable (recall performance), we aimed to create equivalent groups in terms of working memory capacity. Working memory is a critical cognitive resource that influences learning, memory encoding, and retrieval (Baddeley, 2000; Sweller, 1988). Research has demonstrated that individual differences in working memory capacity can significantly affect performance, particularly in memory-related tasks (Greener, 2017). While random assignment is generally sufficient in large samples, our sample size (*N* = 60) made it necessary to balance this variable explicitly to reduce variability caused by cognitive differences and ensure that group differences in performance could be attributed solely to the experimental conditions. This approach aligns with best practices in experimental research for controlling confounding variables (Kirk, 2013).

The working memory capacity test results of the selected groups are given below.

The results presented in Table 1 and Table 2 indicate that there was no statistically significant difference in working memory capacity between the groups. The cognitive capacities of the groups were balanced and they had similar levels of working memory capacity before the experimental intervention according to the Kruskal–Wallis H test results (*p* > 0.05). This result indicates that the performance differences observed in the experimental phase were due to the interventions (observation, photo-taking, or video recording) rather than changes in initial working memory capacity.

### 2.2. Materials

First, the objects exhibited in the museum were examined by the researchers, and it was determined that they were in three main categories: oil paintings, ship models and tools and equipment removed from the inventory. Four objects from each of the determined categories were selected for the experiment. The four oil paintings selected were among the larger sizes among the other paintings in the museum. In the model category, ship models with larger sizes were selected in order to make them easier to examine. The tools and equipment removed from the inventory were four of the five objects exhibited in the garden of the museum. The fifth object was a coast guard helicopter and was recently removed from the inventory. The helicopter was not included in this category because the participants might have seen the helicopter before.

### 2.3. Data Collection Tools

Working Memory Test: The computer-based “Numerical Memory Updating Subtest of the WMC Test” selected by Lehmann et al. (2015) and developed by Oberauer et al. (2000) was used to assess working memory capacity. The highest score to obtain from this test is 15, and the lowest score is 0.

Recall Test: A recall test was developed by the researchers to determine what the participants recalled after the museum visit. A total of 12 open-ended questions were prepared within the scope of the test, four from each category of oil paintings, ship models and materials removed from the inventory. In order to find the content validity rates of these questions, the opinions of ten experts were obtained using the Ayre and Scally (2014) technique. In the Ayre and Scally (2014) technique, the critical Content Validity Criterion alpha value accepted for ten experts is 0.62. Since the content validity index calculated within the scope of the study is 0.89, it is thought that the developed questions have the validity to be applied in the research. In the recall test, participants were given 1 point (for a piece of content) for each point they recalled about the objects in the question.

### 2.4. Ethical Process

The ethical dimension of this research was carried out with the permission of Mersin University Social and Human Sciences Ethics Committee dated 5 April 2024 and numbered 85. The studies were conducted in accordance with local legislation and institutional requirements. Participants gave written informed consent to participate in this study. Written informed consent was obtained from the individual(s) for the publication of potentially identifiable data in this article.

### 2.5. Procedure

After the study groups were determined, the participants (each group on a different day) were taken to the Maritime Museum in Mersin and toured the museum. The participants were asked to visit the museum according to the following criteria and make observations. After the tour, the participants were told that a short recall test would be administered.

Group 1: The museum would only be visited and observed. Photography and video recording was prohibited.

Group 2: The museum would be visited. Only photography would be allowed during the tour.

Group 3: The museum would be visited. Only video recording would be allowed during the tour.

#### 2.5.1. Research Process with Observing Group

Participants were given the opportunity to observe each object for 15 uninterrupted seconds, a duration consistent with the research procedures employed by Schäffers (2022) and Soares and Storm (2018). Furthermore, prior studies by Hollingworth and Henderson (2002), Hyun and Luck (2007), Rensink et al. (1997) and Levin and Simons (1997) have demonstrated that a 15-s visual observation enhances attention and visual memory, facilitating the effective encoding of information into long-term memory. They were instructed to ‘focus on the objects for the duration’ before beginning the experiment process. This instruction given to the participants structured the observation process by allowing them to focus their attention on the objects. Such focusing guidance helps more effective encoding of information into memory by preventing unnecessary cognitive load (Chandler & Sweller, 1991; Schäffers, 2022). When the object’s observation time was over, the participants were directed to the next object belonging to the same category. After all 12 objects were observed, tablets were distributed to the participants and they were allowed to play the Tetris game for ten minutes. At the end of the period, a recall test was distributed to the them. After the participants were informed about the content and purpose of the recall test consisting of a total of 12 questions, they were given 30 min to complete the test. They were also asked to fill in their demographic information at the end of the test within 5 min. At the end of the period, the participants were thanked and the tests were collected.

#### 2.5.2. Research Process with Photo-Taking Group

Before the experiment, they were given time to practice using their camera application on their smartphones and told to be sure to line up the shot carefully via angling the camera vertically or horizontally and also zooming in as needed to obtain best shot of the objects. They were instructed to ‘take a photo of each object with the camera application and save them in the photo albums on their smartphones.’ and ‘continue to look at the object for the rest of the 15 seconds’. The rest of the process were carried out in the same way as those in the observing group. Participants were given the opportunity to photograph each object for 15 uninterrupted seconds for the reasons (Hollingworth & Henderson, 2002; Hyun & Luck, 2007; Rensink et al., 1997; Schäffers, 2022; Levin & Simons, 1997; Soares & Storm, 2018) as mentioned above (in the Section 2.5.1). On the other hand, in the studies of Henkel (2014); Soares and Storm (2018), participants were given time to observe the objects after photo-taking, and this was also thought to be appropriate for the nature of the act of taking photographs in real life, so the same process was applied in this paper.

#### 2.5.3. Research Process with Video-Recording Group

Participants were given the opportunity to videotape each object for 15 uninterrupted seconds for the reasons (Hollingworth & Henderson, 2002; Hyun & Luck, 2007; Rensink et al., 1997; Schäffers, 2022; Levin & Simons, 1997; Soares & Storm, 2018) mentioned above (in the Section 2.5.1) as in the photo-taking group. They were instructed to ‘record videos as they do on their phones in daily life, but the entire object must be in the frame during recording’ and not to ‘use the phone’s zoom function’ before beginning the experiment process. The rest of the process was carried out in the same way as in the observation group and the photo-taking group.

#### 2.5.4. The Running Process of the Second Memory Test

The same recall test (delayed recall) was run to the same participants in the three groups two weeks later to assess long-term memory retention. This two-week period is a widely accepted appropriate interval for assessing memory consolidation and long-term memory performance in memory research (Roediger & Butler, 2011). This period provides ample opportunity for memory consolidation while remaining within a practical time frame for assessing long-term memory (Soderstrom & Bjork, 2015), allowing for a comprehensive analysis of the effects of digital media use on both short-term learning and long-term memory.

Participants did not receive any feedback regarding their performance on the immediate recall test. This was intentionally designed to prevent potential learning effects or performance improvements between the two testing sessions. By withholding feedback, this study ensured that the observed differences in delayed recall were attributable solely to the experimental conditions (observation, photo-taking, and video recording) without introducing confounding variables that could bias the results.

All photographic and video recordings were made using the participants’ personal smartphones. However, following the completion of the first recall test, participants in both the photo-taking and video-recording groups were explicitly instructed to delete all relevant files (photos/videos) from their devices. The deletion process was conducted in the presence of the research team, who confirmed and documented the removal of the files. This procedure was implemented to eliminate potential confounding factors, such as post-visit review or cueing effects, which could arise from participants revisiting the digital files during the two-week delay. Participants were informed of this protocol prior to the study, and compliance with these instructions was ensured to maintain the integrity of the experimental conditions.

### 2.6. Data Analysis

In order to score the participants’ responses to the recall test, the details in the works were defined in advance. Accordingly, 1 point was given for each detail that was remembered correctly about the materials and that could be seen in the table, while no points were given for incorrect answers. Expressions that did not include details about the materials (e.g., expressions such as “There was a sea.”, “The ships were fighting.”, “The color blue was used in the table.” for the “Battle of the Sheep Islands” table) were not scored.

## 3. Results

Since normality tests can give misleading results in groups under 30 (Razali & Wah, 2011) and the number of participants in the groups in the study was less than 30, it was assumed that the normality condition was not met, and a non-parametric test was used (Efron & Tibshirani, 1994). The Kruskall–Wallis H test was applied to investigate whether the scores obtained from the first recall test differed according to the groups.

Table 3 shows that scores of the groups in the first recall test differ significantly, x^2^ (sd = 2, n = 60) = 27.107, *p* < 0.05. In addition, effect size calculations indicated that this difference was large in magnitude (η^2^ = 0.44; ε^2^ = 0.46), showing that the observed group differences were not only statistically significant but also practically meaningful. This finding reveals that participants have different levels of recall depending on their museum tour. Pairwise comparisons were made to determine which groups caused this difference. As a result of the pairwise comparisons, the following two situations were determined: There is a significant difference between the group that observed and the group that took photos in favor of the group that observed. There is also a significant difference between the group that observed and the group that recorded videos in favor of the group that observed. These results can be interpreted as recall being higher in cases where only observation is deployed and gradually weakening when photo-taking and video recording were deployed.

In order to determine whether there was a significant difference between the first and the second recall test after the intervention applied to the three groups, a nonparametric ANOVA was applied for a 3 × 2 factorial design using the nparLD 2.2 (Noguchi et al., 2012) package in R software (version 4.5.1). Accordingly, the first recall test and the second recall test mean ranks of each group and the relative treatment effect values and the ANOVA results are given in Table 4. The Relative Treatment Effect (RTE) serves as an effect size measure for non-parametric analyses in repeated-measures factorial designs (Brunner et al., 2002). This statistic was operationalized in the nparLD package by Noguchi et al. (2012), facilitating its application in various research contexts. For example, an RTE value of 0.870 for the First Recall Test in Group 1 (Observing Group) indicates an 87% probability that the score of a randomly selected individual from the entire population will be lower than the score of a randomly selected individual from Group 1 (Observing Group) (Noguchi et al., 2012).

Table 4 reveals that the difference between the first recall test and second recall test scores shows a significant difference according to the groups. The results of the pairwise comparisons made to determine which groups showed a significant difference in the treatment effect are given in Table 5. In addition, the RTE values indicate that the effect sizes were large for the observing group (RTE = 0.87), moderate for the photo-taking group (RTE = 0.61), and small-to-moderate for the video-recording group (RTE = 0.50), confirming that the group differences were not only statistically significant but also meaningful in magnitude.

**Figure 1:**
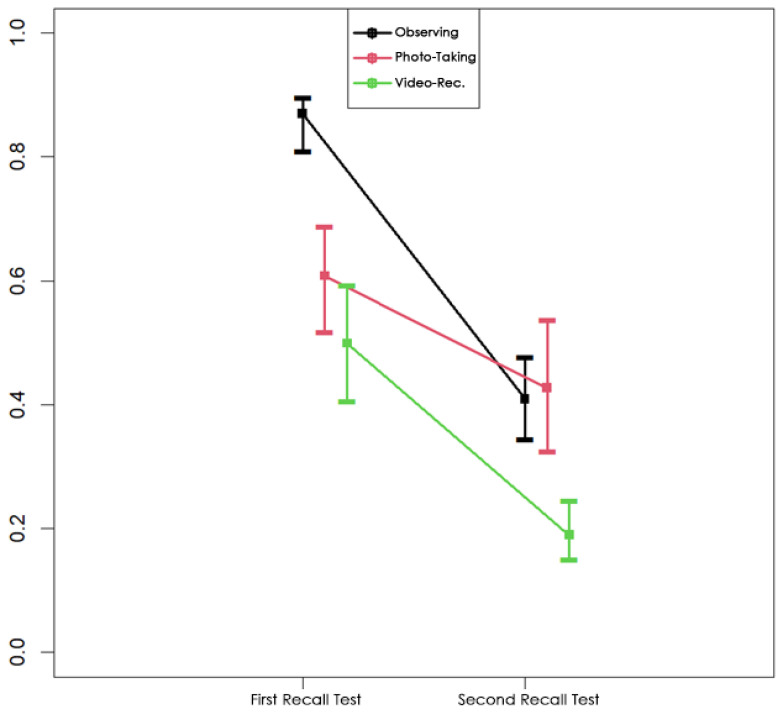
Visualized results.

It is seen that the treatment effect differed significantly between Groups 1 and 2. The relevant results are also visualized in Figure 1.

The findings indicate that in the first recall test conducted immediately after the museum visit, the observation group achieved higher scores compared to the other groups. However, in the second recall test, all groups experienced a decline in their scores. As shown in Table 4 and Table 5 and Figure 1, the extent of this decline varied across groups. The observation group exhibited the largest loss in recall, with a decrease of 78.76%. This can be attributed to their initially higher recall scores, which gave them more information to lose over time. In contrast, the photo-taking group experienced the smallest loss in recall at 39.86%, suggesting that photographs may have served as cues aiding long-term memory retention. The video-recording group showed a loss of 78.10%, similar to the observation group, and performed the worst in both immediate and delayed recall tests.

These results highlight that while the observation and video-recording groups experienced similar high levels of recall loss (78.76% and 78.10%, respectively), the photo-taking group demonstrated a significantly lower decline (39.86%). This lower loss in the photo-taking group may indicate that taking photographs, although initially distracting, can support long-term memory retrieval by providing visual cues. On the other hand, the video-recording group’s poor performance across both tests may be attributed to the higher cognitive load and sustained attention required during video recording. As visualized in Figure 1, the video-recording group’s recall scores in the second test were markedly lower than those of the other groups.

These findings suggest that the observation group’s higher initial recall scores led to a greater relative loss over time, while the photo-taking group benefited from the use of photographs as retrieval aids, resulting in lower memory loss. The video-recording group, however, suffered significant losses due to the cognitive demands of the video-recording process, which negatively impacted both short-term and long-term memory performance.

## 4. Discussion

This paper examines the effects of digital media use, particularly photo-taking and video recording, on memory in the context of museum education. The first test results strongly corroborate the photo-taking–impairment effect proposed by Henkel (2014) and developed by Soares and Storm (2018). These results indicate that participants who merely observed the objects had significantly higher memory retention than those who took photographs or record videos. This underscores the critical impact of digital technologies on cognitive processes and their potential to disrupt learning experiences. On the other hand, cognitive offloading should not be considered merely as a disruptive process. Some studies show that transferring cognitive tasks to external devices can free up individuals’ cognitive resources and these resources can be directed towards higher-level cognitive activities such as interpretation, discrimination and reflective thinking (Sparrow et al., 2011; Risko & Gilbert, 2016). From this aspect, although taking a photo and video recording limits the recall of superficial details, it can also have a function of supporting deep cognitive engagement with purposeful and pedagogical guidance, and the results of the second recall test showed that the photo-taking group had better long-term memory performance than the other groups. This finding suggests that taking photos can support memory by providing clues in the long-term recall process and reveals both positive and negative aspects of the photo-taking effect on memory (Fawns, 2022; Sparrow et al., 2011). Video recording has been shown to negatively affect both short- and long-term memory processes due to high cognitive load (Diehl & Zauberman, 2022; Sweller, 1988).

### 4.1. Cognitive Offloading and Memory Impairment

The results of this study regarding the negative effects of taking photos and videos on recall clearly align with the cognitive offloading theory (Risko & Gilbert, 2016), which posits that when individuals rely on external devices like smartphones, cameras or similar devices, they offload cognitive resources onto these devices, thus reducing the mental effort required for memory encoding. As Henkel (2014) stated, despite the attention required to angle the camera and adjust the lens for the purpose of capturing the best shot of the object completely, the act of photographing the object may enable people to erase the object from their memory, thereby relying on the external device of the camera to “remember” for them. A similar situation is also seen in the cognitive process that Sparrow et al.’s (2011) also called the ‘Google effect.’ This offloading can lead to diminished retention, as participants may subconsciously reduce the attention allocated to the experience itself, knowing that the device will store the information (Sparrow et al., 2011). Murphy (2023) supports this view by demonstrating that offloaded information is often forgotten when the external device becomes unavailable. In a study conducted by Murphy (2023) supporting these results, it was emphasized that when information is transferred to external devices, it is often forgotten when these devices cannot be accessed. This finding reveals that cognitive offloading affects not only the storage of information but also the processes of accessing information.

The observation group had the highest scores in the short-term memory test compared to the other groups of this study, remarking that digital devices can disrupt memory encoding processes. However, the reduction in performance of the photo-taking group was more limited than the other groups in the second recall test, suggesting that photos may serve as a cue for long-term memory (Fawns, 2022). Photographs ease the process of recalling specific visual details but may prevent the contextual and holistic recall of the experience (Tamir et al., 2018). Therefore, contrary to Soares and Storm’s (2018) view that taking a photo prospectively disrupts how an experience is encoded, even if the object is continued to be viewed after the phone is put down, it can be said that taking a photo can help in recalling information from a long-term memory perspective.

Although it is stated by both Cognitive Load Theory (Chandler & Sweller, 1991) and the Cognitive Theory of Multimedia Learning (Mayer et al., 2001) that the same or unnecessary information creates an additional load on memory and hinders learning, a literature review on this subject has shown that repeating the content can be beneficial for students who have no prior knowledge on the subject from the aspect of the redundancy effect. On the other hand, it has been determined that it hampers learning on its own for students with high levels of prior knowledge about the learning subject. Considering that the visitors in this paper saw the objects in the museum for the first time, they therefore had little or no prior knowledge, and it is thought that the repetition of information about the objects through different channels (viewing the objects both visually and through the smartphone screen) may have been effective in the fact that the participants in the photo-taking group had less loss in the long-term memory test compared to the other groups. However, due to unnecessary information flow from the camera application and other stimuli around the objects during photo-taking, the level of short-term recall may be lower than the level of long-term recall.

On the contrary, the video-recording group performed the lowest on both short- and long-term memory tests. This may be explained by the fact that video recording requires perpetual attention, and so it creates too much cognitive load (Chandler & Sweller, 1991; Murphy, 2023). Additionally, in Soares and Storm’s (2018) study, participants in the Snapchat condition recalled fewer details because they interacted with their phones more than those who took photos. Similarly, the lower performance of the video-recording group in this paper in terms of both short-term and long-term memory may have been due to the longer time they spent interacting with their phones compared to the photo-taking group. Just like the photo-taking group, the participants in the video-recording group may have obtained these results due to the unnecessary flow of information from the camera application and other stimuli around the objects in addition.

### 4.2. Photo-Taking–Impairment Effect and Selective Attention

‘Photo-taking–impairment effect’ put forward by Henkel (2014) suggests that participants were not focused on the experience in detail while photography and therefore had difficulty handling details. This phenomenon can also be clarified by Craik and Lockhart’s (1972) ‘selective attention theory’. According to the theory, individuals assign cognitive resources to the stimuli they perceive according to their order of importance. While the participants who took photographs in this paper focused on capturing the image, they may have been incapable of fully processing the contextual and visual details of the objects. This is consistent with the suggestion by Tamir et al. (2018) that media usage can reduce the accuracy and integrity of autobiographical memory.

Although taking photos had a distracting effect on the short-term memory test (first test), there was less of a decrease in the long-term test (second test) in this study. These results are coincide with research revealing that taking photos may facilitate the processes of recalling specific details but that the experience may reduce the overall quality of remembering (Barasch et al., 2017). Conversely, the video-recording group experienced a higher extraneous load, which negatively affected memory processes. This is thought to be through the perpetual attention and management requirements outlined by Diehl and Zauberman (2022).

### 4.3. Implications for Educational Environments

This paper suggests that the integration of digital media tools into educational environments should be handled meticulously. Effective learning requires individuals to interact with material actively and meaningfully according to ‘constructivist learning theory’ (Piaget, 1954) and Vygotsky’s (1978) ‘sociocultural learning approaches’. Therefore, the effects of digital devices should not be considered as strictly positive or negative. Their effects on learning processes depend on how and for what purposes they are pedagogically integrated. For instance, taking a photo can serve as an external cue that facilitates later recall, while intense and uninterrupted video recording can increase cognitive load and lead to distraction (Diehl & Zauberman, 2022; Wong & Lim, 2023). Therefore, conscious and balanced guidance of digital devices can support the comprehensive learning objectives of the museum education. However, the findings also indicate a potential redundancy effect due to the simultaneous recording and observation process. Observing the background simultaneously with photo-taking and video recording required processing across multiple channels, which may have created an additional cognitive load and had a negative impact on learning. Human working memory has limited capacity, and when individuals are required to divide their attention between experiencing an event and operating a recording device, their ability to encode and retain information decreases as revealed by ‘cognitive load theory’ (Chandler & Sweller, 1991). Furthermore, Ophir et al. (2009) demonstrated that multitasking diminishes cognitive control and attentional capacity, which negatively impacts learning outcomes. On the other hand, guided observation can be considered as a strategy of thought in balancing cognitive demands because the structured guidance helps learners focus attention on relevant information, thus reducing unnecessary burden (Kirschner et al., 2006; Moreno & Mayer, 2007).

Mayer’s (2005) ‘cognitive theory of multimedia learning’ claims that digital media is effective only when used to promote learning materials. Similarly, Diehl and Zauberman (2022) suggested that digital devices can increase focus when used mindfully, but too much or unplanned use can negatively affect learning processes. This result is in line with the superior performance of the observation group in the first test and the learning differences in the groups using digital media.

According to Hutchins’ (2000) distributed cognition perspective, digital devices can lighten individuals’ cognitive load, but at the same time, they can also alter information processing processes. The results of this study show both positive and negative effects of digital devices. It can be seen that taking photographs can contribute to the process of remembering visual details, but it can limit the capacity to process the overall context of the experience. On the other hand, video recording caused a significant decrease in learning outcomes due to the need for sustained attention. This situation emphasizes that digital devices should be carefully integrated as a tool to support learning.

The results obtained indicate the importance of a conscious and balanced integration of digital tools in the design of educational environments. In experiential learning environments such as museums, strategies that encourage the active participation of participants and ensure that digital tools are used only as a tool to support learning should be developed. These approaches can increase deep cognitive engagement in learning processes and contribute to the improvement of learning outcomes while minimizing the distracting effect of digital media.

## 5. Suggestions

The findings of this study highlight the importance of integrating digital media into educational processes in a conscious and balanced manner. Researchers should examine the effects of digital tools on cognitive load and learning more comprehensively, especially analyzing the different effects of taking photos and recording videos. Educators should encourage participants to engage more deeply with learning materials by providing guidance that limits the distracting effects of digital media.

Educational environments should be designed in a way that digital tools contribute to the learning process while maintaining the focus of attention. In experiential learning environments such as museums, strategies that increase the active participation of participants should be prioritized. These approaches can contribute to both improving learning outcomes and using digital technologies more effectively.

In this context, structuring the use of digital devices and making it purposeful can ease the cognitive load. Based on the findings of this paper and other research, the following can be performed (Cahill et al., 2011; Hughes & Moscardo, 2017; Stylianou-Lambert, 2017): Educators can provide students with clear purpose and guidance on experiences such as museum tours; for instance, they can put them in the role of a curator or ask them to prepare materials for specific audiences. Learning experiences can be organized by project-based approaches, key questions, sub-questions and labels to be created by students via guiding the data collection process. These structures serve as predecessor organizers that help students connect their discoveries to the curriculum. The integration of digital devices into the exhibition design (e.g., designating photo-taking spots in museums for certain objects or messages) can also support this structuring.

## 6. Conclusions

This study examined the effects of digital media tools on learning processes and presented important findings in terms of both short-term memory performance and long-term memory retention. The potential of taking photographs to provide long-term memory cues, despite causing short-term distraction, suggests that these tools should be used consciously in learning environments. In contrast, the high cognitive load of video recording negatively affects both types of memory, emphasizing the need for careful use. The findings/results demonstrate that digital integration in the museum context carries both an opportunity and a limitation. Memory processes are essential not only for remembering objects but also for maintaining cultural continuity, reflective thinking, and critical analyzing (Falk & Dierking, 2013; Hein, 1998). Therefore, the conscious and balanced use of digital devices can strengthen the educational role of museums.

In conclusion, the balanced use of digital tools in educational environments can both increase the active participation of participants and improve the quality of learning outcomes. Future research should focus on developing methods for more effective integration of these tools into educational processes.

Additionally, in this paper, there is no condition where participants can decide on their own whether to take photo/record video or not. Recent studies have shown that taking photos when participants can decide for themselves which objects to photograph (i.e., “prompted photography”) can increase both engagement in an experience (Diehl et al., 2016) and memory about that experience (Barasch et al., 2017). Therefore, it is thought that research should be conducted on how short- and long-term recall will occur when participants are given the choice of whether to take a photo/record a video or not.

Finally, within the scope of Cognitive Load Theory, it would be useful to conduct studies examining whether both photo-taking and video recording create an unnecessary channel when viewing objects in terms of the redundancy effect.

## Figures and Tables

**Table 1:** Descriptive statistics of WMC test results of selected groups.

	N	Min	Max	x	sd
Group 1 (Observing)	20	3	12	9.35	2.82
Group 2 (Photo-Taking)	20	3	12	9.75	1.92
Group 3 (Video-Recording)	20	4	11	9.60	2.33

**Table 2:** WMC test and Kruskal–Wallis H test results of selected groups.

	N	Mean Rank	sd	x^2^	*p*	Diff.
Observing	20	31.08	2	0.036	0.982	-
Photo-Taking	20	30.33				
Video-Recording	20	30.10				

**Table 3:** Kruskal–Wallis H test results of the scores of the groups in the first recall test.

	N	Mean Rank	sd	x^2^	*p*	Diff.
Observing	20	46.60	2	27.107	0.00	1–2, 1–3
Photo-Taking	20	25.80				
Video-Recording	20	19.10				

Note: Effect size estimates were η^2^ = 0.44 and ε^2^ = 0.46, both indicating a large effect.

**Table 4:** Descriptive statistics and nonparametric ANOVA results of the groups.

	First Recall Test			Second Recall Test		
Group No	N	Mean Rank	Relative Treatment Effect	n	Mean Rank	Relative Treatment Effect
1	20	104.85	0.870	20	49.55	0.409
2	20	73.42	0.608	20	51.68	0.426
3	20	60.35	0.498	20	23.15	0.189

F_1.74, 44.00_ = 22.90; *p* = <0.001.

**Table 5:** Group pairwise comparison results for measurement.

Group A–Group B	t	sd	*p*
1–2	8.57	1	<0.001
1–3	3.57	1	0.059
2–3	1.49	1	0.222

## Data Availability

The raw data supporting the conclusions of this article will be made available by the authors, without undue reservation.
